# Supplementary material for: Cilia-driven surface currents characterize specific cnidarian groups and lifecycle stages
Source: Commun Biol. 2026 Mar 11;9:579. doi: 10.1038/s42003-026-09827-0 (PMC13111671; doi:10.1038/s42003-026-09827-0)
Supplement: Supplementary file 2 — Supplementary Information [file 42003_2026_9827_MOESM2_ESM.pdf]

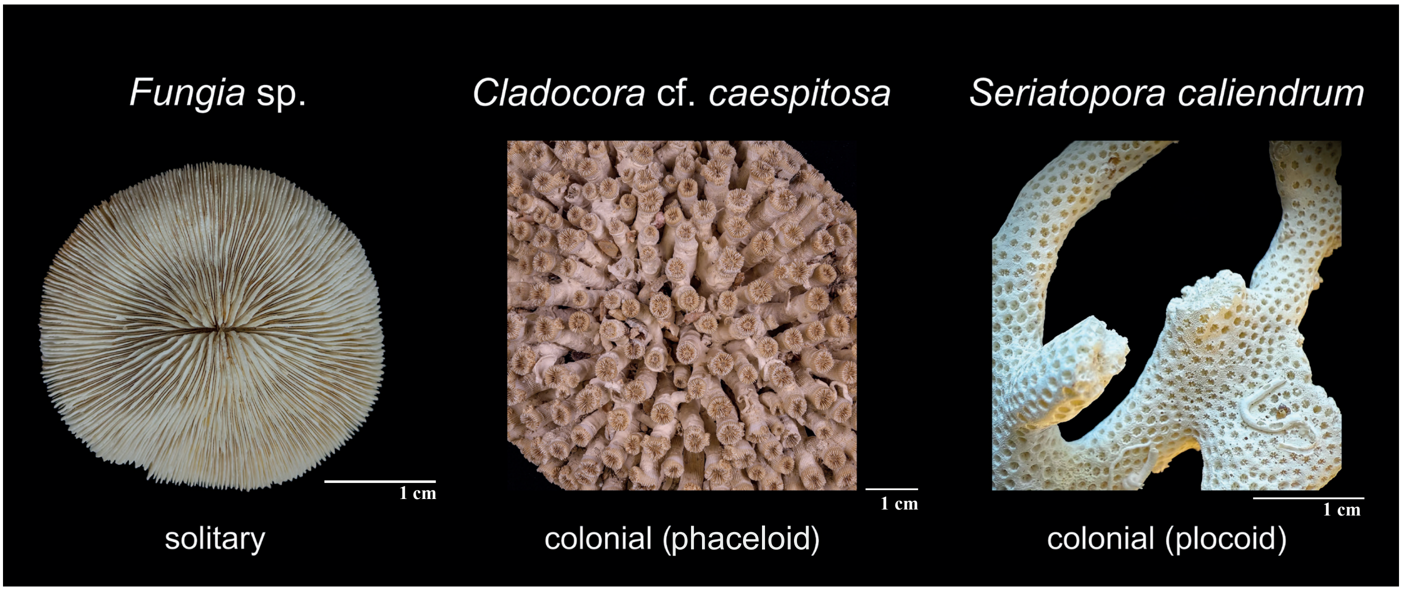

Supplementary Figure 1. Examples of three coral species with different numbers and sizes of polyps. Note the range starting with a large solitary polyp (*Fungia sp.*), going through several connected medium-size polyps of *Cladocora caespitosa*, and finishing with highly colonial *Seriatopora caliendrum* made of hundreds or thousands of miniaturized polyps.

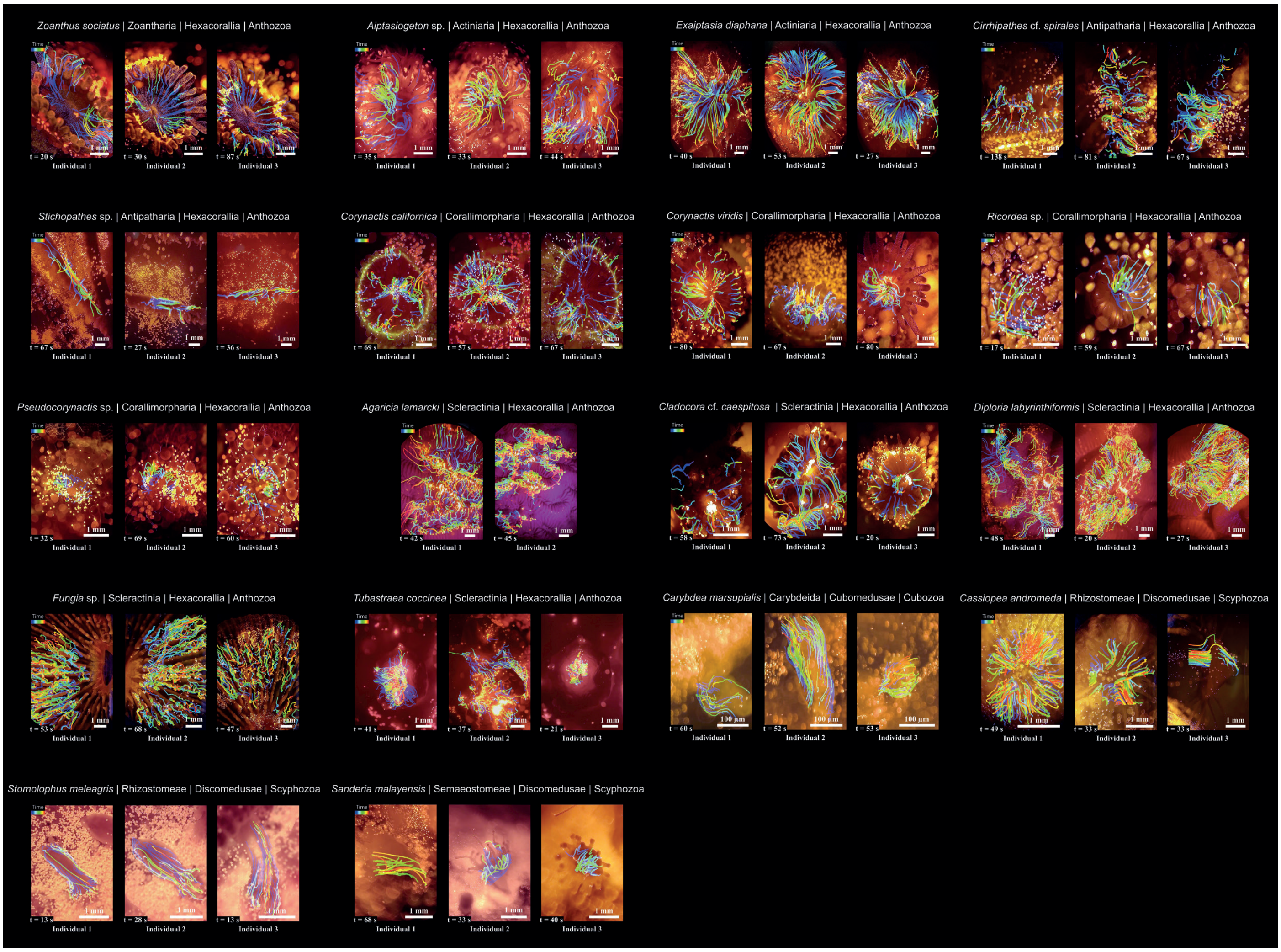

Supplementary Figure 2. Summary of the flow trajectories in species with surface currents. The recorded trajectories result from tracking fluorescent microbeads 100 micrometers in diameter and visualizing them using IMARIS. t denotes the duration over which the beads were tracked, which varied depending on how long it took for the beads to be moved by an animal.

Distribution of **cilia** on the external and internal surfaces of juvenile *Cassiopea andromeda* medusa (post-ephyra stage)

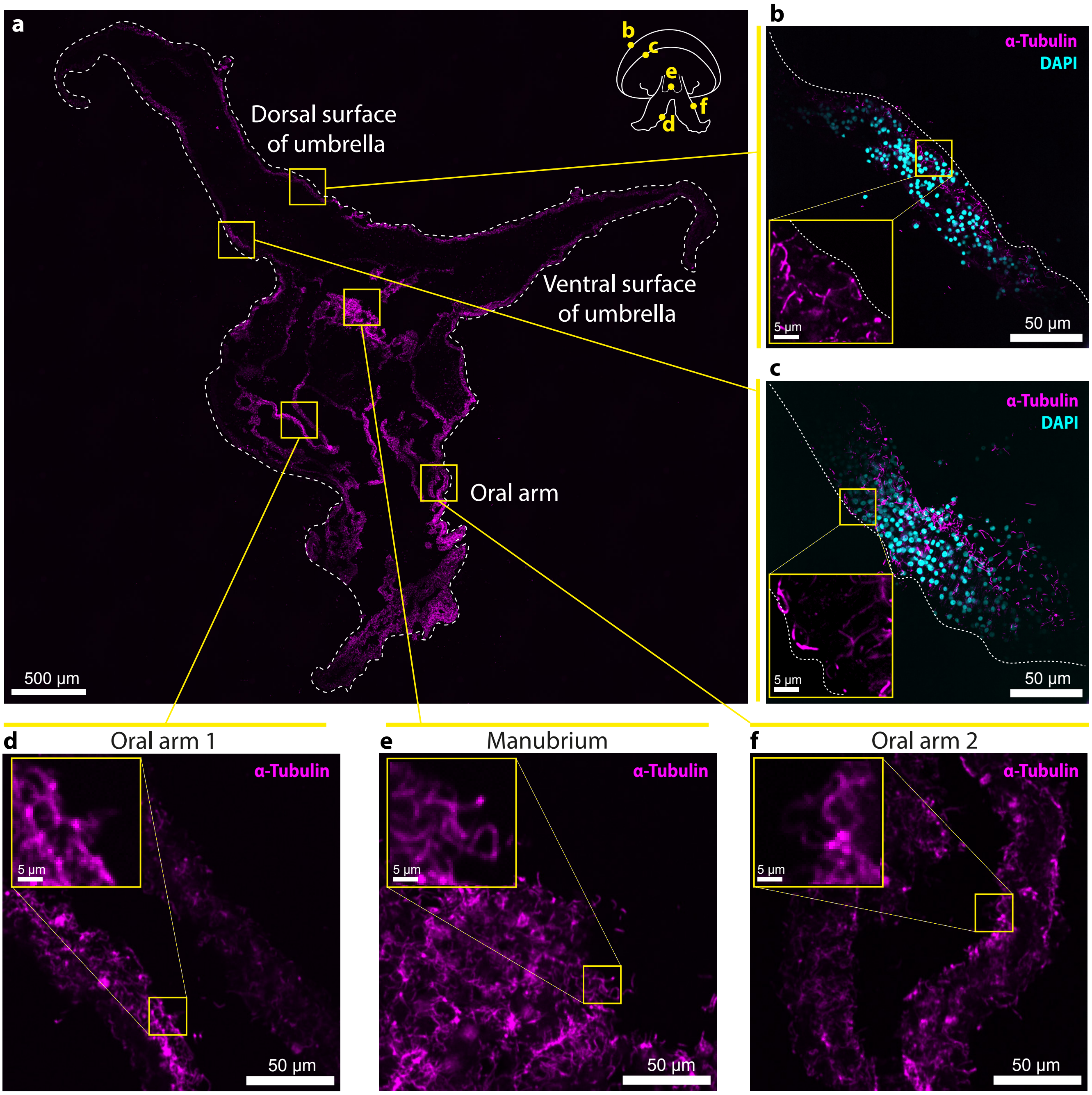

Supplementary Figure 3. Cilia coverage of various anatomical structures in a juvenile *Cassiopea andromeda* medusa. Cilia are stained with anti-α-Tubulin antibodies (magenta). White dashed lines indicate the outer surface of epithelia. (a) Cross-section of the entire juvenile *Cassiopea andromeda* medusa, general view. Yellow frames in (a) indicate positions of the magnified regions: (b-c) ciliation of the dorsal (b) and ventral (c) surfaces of a jellyfish umbrella; (d) ciliation of the oral arm (magnified region 1); (e) ciliation of the manubrium; (f) ciliation of the oral arm (magnified region 2). Scale bars are 50 micrometers.

Stomolophus meleagris *Aurelia coerulea*

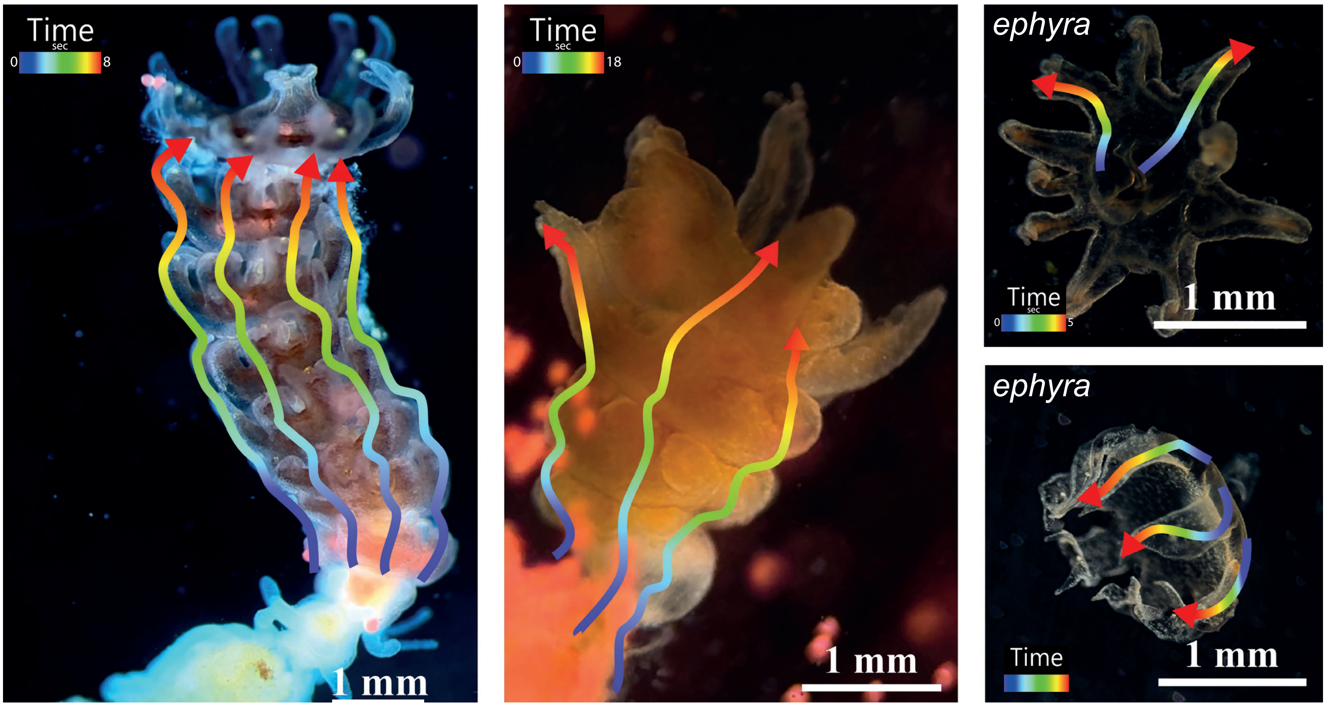

Supplementary Figure 4. Generation of surface currents by scyphozoan strobilae and ephyrae of *Stomolophus meleagris* and *Aurelia coerulea*. Arrows indicate tracks of individual beads. Note the consistent movement of beads from the base/aboral side towards apical/oral side.

Distribution of cilia on the external surface and rhopalium of juvenile *Tripedalia cystophora* medusa

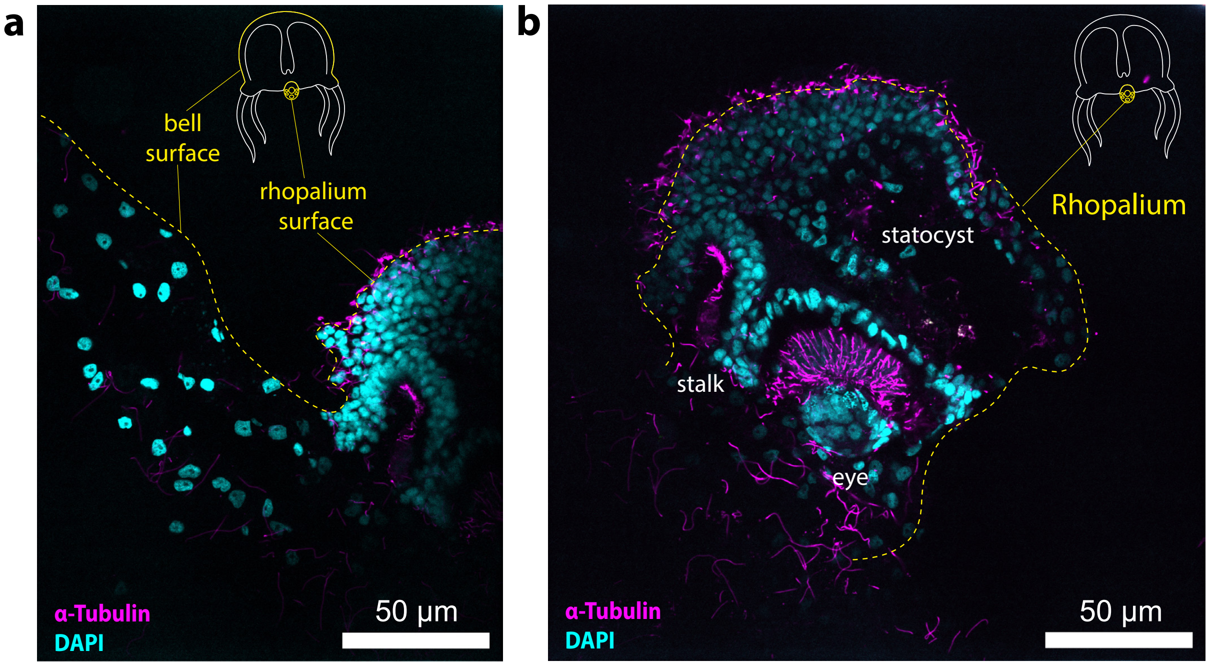

Supplementary Figure 5. Distribution of cilia on the surface of *Tripedalia cystophora* medusa stage. Cilia are stained with anti-α-Tubulin antibody (magenta). White dashed lines indicate the outer surface of epithelia. (a) Cross-section of the bell with rhopalium starting on the left. (b) Cross-section through rhopalium. Note the higher number of cilia covering the rhopalium in comparison with neighboring bell surface area. Scale bars are 50 micrometers.

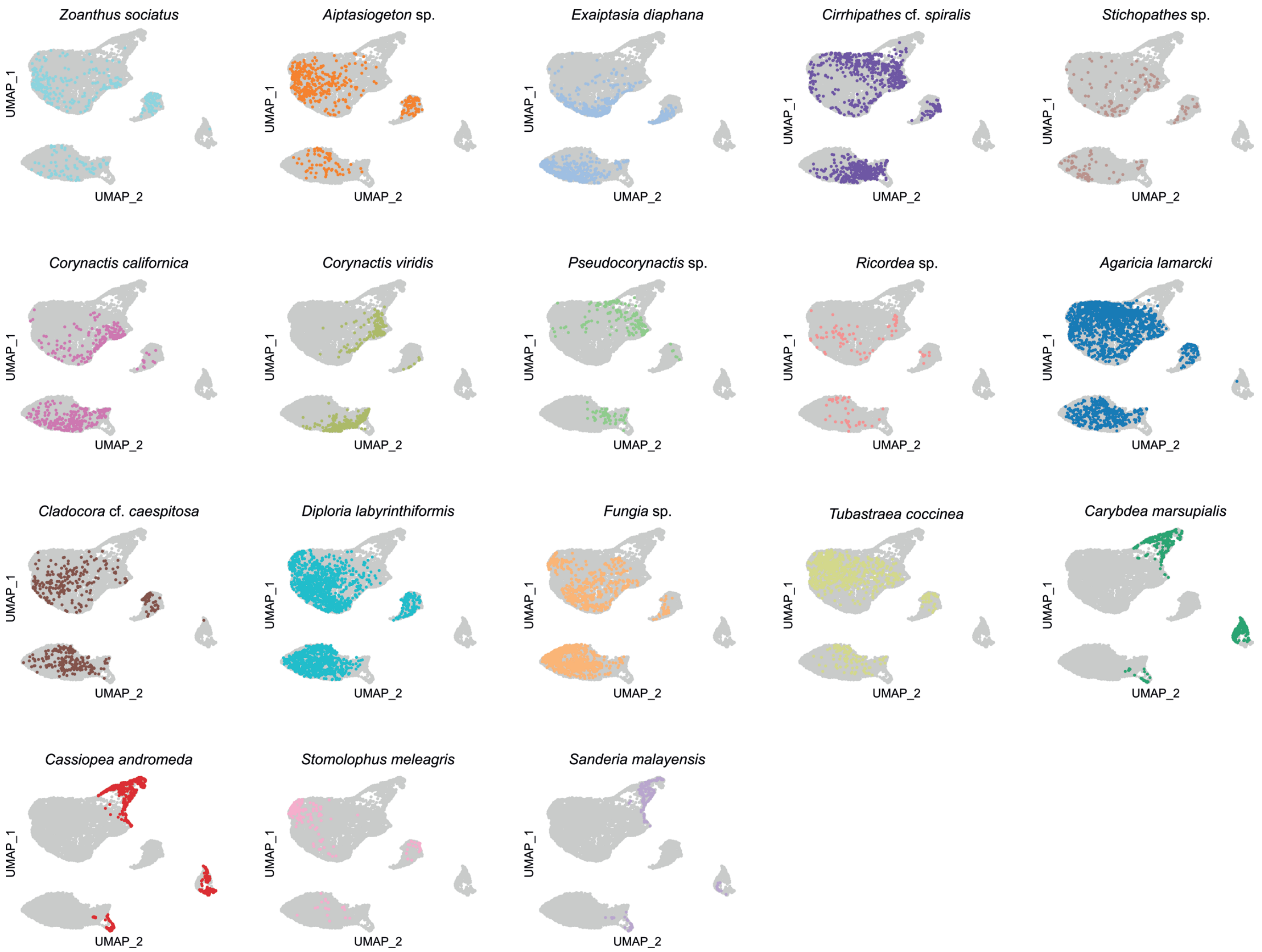

Supplementary Figure 6. UMAP projections and clustering of all investigated species exhibiting surface currents.
