# Supplementary material for: Cilia-driven surface currents characterize specific cnidarian groups and lifecycle stages
Source: Commun Biol. 2026 Mar 11;9:579. doi: 10.1038/s42003-026-09827-0 (PMC13111671; doi:10.1038/s42003-026-09827-0)
Supplement: Supplementary file 3 — Description of Additional Supplementary File [file 42003_2026_9827_MOESM3_ESM.pdf]

Description of Additional Supplementary Files

File name: Supplementary Movie 1

Description: Examples of video recordings of cnidarians with surface currents and without surface currents.

File name: Supplementary Movie 2

Description: Recordings of surface currents generated by different life stages of the scyphozoans *Stomolophus meleagris* (strobila) and *Aurelia coerulea* (strobila / ephyrae).

File name: Supplementary Data 1

Description: Exact numbers of analyzed bead tracks for the studied species and groups

File name: Supplementary Data 2

Description: Key reagents, resources, software, and data
