# Supplementary material for: Cilia-driven surface currents characterize specific cnidarian groups and lifecycle stages
Source: Commun Biol. 2026 Mar 11;9:579. doi: 10.1038/s42003-026-09827-0 (PMC13111671; doi:10.1038/s42003-026-09827-0)
Supplement: Supplementary file 8 — Reporting Summary [file 42003_2026_9827_MOESM8_ESM.pdf]

nature portfolio

Corresponding author(s): Igor Adameyko

Last updated by author(s): Jan 30, 2026

Reporting Summary

Nature Portfolio wishes to improve the reproducibility of the work that we publish. This form provides structure for consistency and transparency in reporting. For further information on Nature Portfolio policies, see our Editorial Policies and the Editorial Policy Checklist.

Statistics

For all statistical analyses, confirm that the following items are present in the figure legend, table legend, main text, or Methods section.

n/a	Confirmed	
☐	☒	The exact sample size (*n*) for each experimental group/condition, given as a discrete number and unit of measurement
☐	☒	A statement on whether measurements were taken from distinct samples or whether the same sample was measured repeatedly
☐	☒	The statistical test(s) used AND whether they are one- or two-sided<br>*Only common tests should be described solely by name; describe more complex techniques in the Methods section.*
☐	☒	A description of all covariates tested
☐	☒	A description of any assumptions or corrections, such as tests of normality and adjustment for multiple comparisons
☐	☒	A full description of the statistical parameters including central tendency (e.g. means) or other basic estimates (e.g. regression coefficient) AND variation (e.g. standard deviation) or associated estimates of uncertainty (e.g. confidence intervals)
☒	☐	For null hypothesis testing, the test statistic (e.g. *F*, *t*, *r*) with confidence intervals, effect sizes, degrees of freedom and *P* value noted<br>*Give P values as exact values whenever suitable.*
☒	☐	For Bayesian analysis, information on the choice of priors and Markov chain Monte Carlo settings
☒	☐	For hierarchical and complex designs, identification of the appropriate level for tests and full reporting of outcomes
☒	☐	Estimates of effect sizes (e.g. Cohen's *d*, Pearson's *r*), indicating how they were calculated

Our web collection on statistics for biologists contains articles on many of the points above.

Software and code

Policy information about availability of computer code

Data collection	Recordings were performed under a white light or with assistance of fluorescent stereomicroscope (Leica M165 FC) equipped with a CoolLED light source (light intensity 200µmol/m2/s (photosynthetic photon flux)) and a Samsung X23 camera (24 frames per second). Recordings were processed using IMARIS 9.6.0 (https://imaris.oxinst.com/). Immunohistochemistry imaging was done using Nikon Eclipse Ti2-E inverted microscope fitted with Yokogawa W1 spinning disc and Nikon Plan Apo 40x/0.95 /0.11-0.23 WD 0.25-0.17 objective. Aquired images were processed using NIS Elements. Version 5.30.02 and ImageJ.
Data analysis	Code for data analysis can be found here: https://github.com/TBouderlique/cnidarians, https://github.com/LouisFaure/coral_paper

For manuscripts utilizing custom algorithms or software that are central to the research but not yet described in published literature, software must be made available to editors and reviewers. We strongly encourage code deposition in a community repository (e.g. GitHub). See the Nature Portfolio guidelines for submitting code & software for further information.

Data

Policy information about availability of data

All manuscripts must include a data availability statement. This statement should provide the following information, where applicable:

- Accession codes, unique identifiers, or web links for publicly available datasets
- A description of any restrictions on data availability
- For clinical datasets or third party data, please ensure that the statement adheres to our policy

All raw data can be reached by: https://doi.org/10.7910/DVN/IMXADV

Research involving human participants, their data, or biological material

Policy information about studies with human participants or human data. See also policy information about sex, gender (identity/presentation), and sexual orientation and race, ethnicity and racism.

Reporting on sex and gender	NA
Reporting on race, ethnicity, or other socially relevant groupings	NA
Population characteristics	NA
Recruitment	NA
Ethics oversight	NA

Note that full information on the approval of the study protocol must also be provided in the manuscript.

Field-specific reporting

Please select the one below that is the best fit for your research. If you are not sure, read the appropriate sections before making your selection.

☒ Life sciences ☐ Behavioural & social sciences ☐ Ecological, evolutionary & environmental sciences

For a reference copy of the document with all sections, see nature.com/documents/nr-reporting-summary-flat.pdf

Life sciences study design

All studies must disclose on these points even when the disclosure is negative.

Sample size	At least three individuals or more were examined per species with few exceptions: for Millepora alcicornis, Agaricia lamarcki, and Gorgonia ventalina, only two colonies were available; Cerianthus sp. was represented by a single specimen. For the recordings of Scyphozoan strobilae, we used two Stomolophus meleagris and two Aurelia coerulea strobilae. Sample size was usually determined for each species by the availability of alive animals.
Data exclusions	No samples were excluded.
Replication	Often, the same individuals were recorded or observed multiple times to ensure the consistency of data prior to the analysis stage.
Randomization	NA
Blinding	NA

Reporting for specific materials, systems and methods

We require information from authors about some types of materials, experimental systems and methods used in many studies. Here, indicate whether each material, system or method listed is relevant to your study. If you are not sure if a list item applies to your research, read the appropriate section before selecting a response.

Materials & experimental systems

n/a	Involved in the study
☐	☒ Antibodies
☒	☐ Eukaryotic cell lines
☒	☐ Palaeontology and archaeology
☐	☒ Animals and other organisms
☒	☐ Clinical data
☒	☐ Dual use research of concern
☒	☐ Plants

Methods

n/a	Involved in the study
☒	☐ ChIP-seq
☒	☐ Flow cytometry
☒	☐ MRI-based neuroimaging

Antibodies

Antibodies used	mouse anti-acetylated tubulin, Abcam, ab24610
Validation	Abcam, ab24610 antibody was validated for mouse, rat, human and cow by manufacturer (https://www.abcam.com/en-us/products/primary-antibodies/alpha-tubulin-acetyl-k40-antibody-6-11b-1-ab24610). It was also validated for coral Stylopfora pistillata (Tambutté et al., 2020, DOI: 10.1007/s00441-020-03343-1)

Animals and other research organisms

Policy information about studies involving animals; ARRIVE guidelines recommended for reporting animal research, and Sex and Gender in Research

Laboratory animals	NA
Wild animals	Aiptasiogeton sp., Anemonia cf. manjano, Anemonia viridis, Anthopleura elegantissima, Exaiptasia diaphana, Cirrhipathes cf. spiralis, Stichopathes sp., Corynactis californica, Corynactis viridis, Pseudocorynactis sp., Ricordea sp., Cladocora cf. caespitosa, Fungia sp., Parazoanthus axinellae, Zoanthus sociatus, Cerianthus sp., Guaiagorgia anas, Isis hippuris, Menella sp., Pseudoplexaura sp., Pseudopterogorgia sp., Sinularia sp., Eudendrium sp., Hydractinia sp., Cassiopea andromeda, Nausithoe punctata, Sanderia malayensis, Stomolophus meleagris were reared, maintained, and propagated ex situ at Haus-des-Meeres (HdM), a public marine aquarium in Vienna, Austria. Carybdea marsupialis, Tripedalia cystophora were obtained from the culture at Vienna Zoo, Vienna, Austria. Xenia sp., Pteroeides griseum, Cladonema sp., were maintained and recorded at Pula Aquarium, Pula, Croatia. Craterolophus convolvulus, Alcyonium sp., Ectopleura crocea were recorded at Helgoland Biological Station, Helgoland, Germany. Agaricia lamarcki, Diploria labyrinthiformis, Tubastraea coccinea, Gorgonia ventalina, Stylaster roseus, Millepora alcicornis, and were examined at the CARMABI research station in Curaçao, Southern Caribbean Sea, in September 2022 . The specimens are part of an exchange between the CARMABI Institute (CITES #AN001) and the Natural History Museum Vienna (Naturhistorisches Museum Wien, CITES #AT017). All material was collected in Curaçao (former Netherlands Antilles) under collection permits issued to CARMABI by the Government of Curaçao (permit CARMABI 2019/021824).
Reporting on sex	NA
Field-collected samples	Immediately after collection, specimens or colonies were transferred to the laboratory and placed in glass containers (20 × 10 cm) filled with seawater from their original environment (either in the aquarium or their natural habitat). Each specimen was covered with 1–2 cm of filtered seawater and allowed to acclimate for one to four hours in an artificial light-free environment with provided water current and conditions similar to maintenance environment.
Ethics oversight	NA

Note that full information on the approval of the study protocol must also be provided in the manuscript.

Plants

Seed stocks	NA
Novel plant genotypes	NA
Authentication	NA
